# Gut microbiota, metabolites, and cytokines in relation to the risk of prostate cancer in the Asian population

**DOI:** 10.3389/fonc.2024.1466190

**Published:** 2025-01-15

**Authors:** Zhengshi Wang, Haotian Chen, Yongqiang Liu, Libin Zou, Zhijin Zhang, Zhiqiang Yin, Shiyu Mao, Changcheng Guo, Bin Yang, Pengfei Wu, Xudong Yao

**Affiliations:** ^1^ Department of Urology, Shanghai Tenth People’s Hospital, Clinical Medical College of Nanjing Medical University, Shanghai, China; ^2^ Department of Urology, Shanghai Tenth People’s Hospital, School of Medicine, Tongji University, Shanghai, China; ^3^ Urologic Cancer Institute, School of Medicine, Tongji University, Shanghai, China; ^4^ Department of Breast and Thyroid Surgery, Shanghai Tenth People’s Hospital, Tongji University School of Medicine, Shanghai, China; ^5^ Shanghai Center of Thyroid Diseases, Shanghai Tenth People’s Hospital, Tongji University School of Medicine, Shanghai, China

**Keywords:** prostate cancer, GWAS, gut microbiota, single nucleotide polymorphism, cytokine

## Abstract

**Purpose:**

Studies have shown that gut microbiota is involved in the tumorigenesis and development of prostate cancer. We aimed to perform a comprehensive analysis of causal associations of gut microbiota, metabolites, and cytokines with prostate cancer in the Asian population.

**Patients and methods:**

Genome-wide association study (GWAS) summary datasets were collected from the public databases. There were 418 bacterial traits, 452 metabolites, 91 cytokines, 5408 cases of prostate cancer from East Asia, and 109,347 controls included. Mendelian randomization (MR) analyses were performed to investigate their causal relationships. Sensitivity analyses were conducted to test the reliability of MR results. Furthermore, the FinnGen database was used to assess the generalizability of our findings based on Asians.

**Results:**

There were a total of 17 bacterial traits, 28 metabolites (including 2 microbiota-associated metabolites), and 9 cytokines to be significantly associated with prostate cancer in Asians (P < 0.05). Further MR analyses of these positive results indicated that *G_Ruminococcaceae UCG014*/TNFSF10 axis, *G_Anaerofilum*/TNFRSF14 axis, *G_Erysipelotrichaceae UCG003*/TNFSF10 axis, and P_Proteobacteria/cholesterol axis were key signaling pathways involved in the progression of prostate cancer. Notably, *G_Ruminococcaceae UCG014*/TNFSF10 axis and *G_Anaerofilum*/TNFRSF14 axis were found to act as protective factors, while the other two signaling axes played a crucial role in promoting the progression of prostate cancer. Sensitivity analyses further confirmed the reliability of our findings. Using the European population as outcome, we further assessed the generalizability of our conclusions and found limited applicability to Europeans.

**Conclusions:**

We found that there were causal associations of gut microbiota, metabolites, and cytokines with prostate cancer in Asians. The causal effects of gut microbiota on prostate cancer were partially mediated by metabolites and cytokines. These findings might contribute to the development of new therapeutic strategies for prostate cancer.

## Introduction

Prostate cancer is one of the most common malignancies among men. The Global Cancer Statistics 2020 indicated that there were approximately 1,414,259 new cases of prostate cancer, which were second in frequency, after lung cancer, for men worldwide (1). Patients with early-stage prostate cancer are usually associated with an overall favorable prognosis, while those with advanced prostate cancer exhibit worse clinical outcomes (2, 3). Although androgen deprivation therapy (ADT), the most classic treatment, showed promising therapeutic effects for prostate cancer, 10%-20% of cases can progress into castration-resistant prostate cancer (CRPC) within five years (4). Therefore, there is an urgent need for in-depth understanding the mechanisms of tumorigenesis and development of prostate cancer to develop new therapeutic options.

Accumulating evidence suggests gut microbiota is involved in diverse aspects of tumorigenesis and development of malignancies, including prostate cancer (5–7). Some commensal bacteria (e.g. Ruminococcus sp. DSM_100440) mediated the process of androgen activation, eventually leading to the accelerated development of CRPC (8). Makoto et al. (9) collected swab samples (96 cases and 56 controls) and found that patients with high-risk prostate cancer possessed specific microflora. However, the close relationships between gut microbiota and prostate cancer do not always imply causal relationships. Mendelian randomization (MR) methods provide suitable tools for investigating causal relationships between gut microbiota and prostate cancer. Wang et al. (10) included 211 bacterial traits and large prostate cancer cohorts in the European population, and identified the causal relationships between gut microbiota and prostate cancer. Similarly, Wei et al. (7) also performed the MR analyses using the 211 bacterial traits and genome-wide association study (GWAS) data of prostate cancer, and yielded positive results. Nevertheless, compared with thousands of bacterial taxa, 211 bacterial traits seem relatively few. On the other hand, the identity of 211 bacterial traits is not established down to the species, which poses an obstacle for the accurate understanding the roles of specific bacteria. Recently, the number of bacterial traits was updated to 418 and information on the species level was also added. Therefore, we performed comprehensive MR analyses using the latest GWAS summary data on gut microbiota, metabolites, cytokines, and prostate cancer from East Asia. These would broaden the understanding of the roles of gut microbiota in prostate cancer and contribute to the development of new therapeutic strategies.

## Methods

### Study design

**Figure 1:**
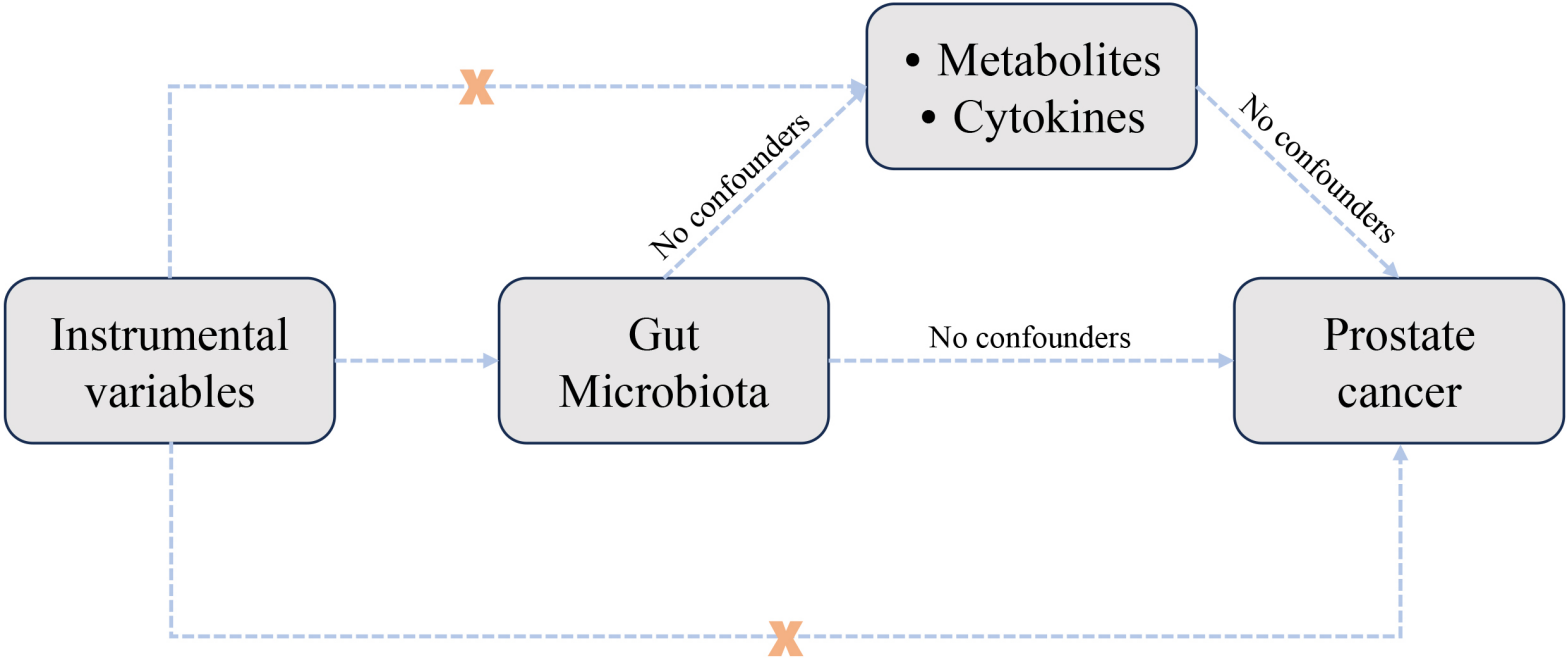
Study design used to investigate the causal relationships between gut microbiota, metabolites, cytokines, and prostate cancer through Mendelian randomization analysis.

The study design was illustrated in **Figure 1**. The MR analysis investigated the causal effects of gut microbiota, metabolites, and cytokines on the risk of prostate cancer. Sensitivity analysis was conducted to assess MR results and ensure their reliability.

### Data sources

The GWAS summary datasets of gut microbiota were downloaded from MiBioGen (https://mibiogen.gcc.rug.nl), which were the most common datasets in the gut microbiota-associated MR analysis. According to taxonomic categories, there were a total of 418 bacterial traits, including 14 phylum, 26 class, 33 order, 58 family, 160 genus, and 112 species. Fifteen bacterial traits with unknown taxonomic categories were excluded. The summary datasets of metabolites were provided by Shin et al. (11). Gut microbiota-associated metabolites were identified using the Human Metabolome Database (HMDB). A total of 91 cytokines were obtained from the previous publication (12), including 11 large cohorts and 14,824 European participants.

To screen valid single nucleotide polymorphisms (SNPs), P-value of genome-wide significance was set as P < 10^-5^ based on the general practice of microbial consortia. To rule out the influence of linkage disequilibrium (LD), two filter conditions were set as follows: (1) the physical distance between two SNPs should be greater than 10,000 kb; (2) The statistical indicator R^2^ for LD should be less than 0.001. To ensure strong relationships between instrumental variables and exposure, SNPs with the F statistic greater than 10 were retained. The F statistic was calculated using the formula: F = (Beta/SE)^2^.

The prostate cancer coded as bbj-a-148 in the IEU OpenGWAS project (https://gwas.mrcieu.ac.uk/) was selected as outcome, which was the unique summary dataset form East Asia. Its sample size is sufficiently large to conduct a genome-wide association study, and the release time (2019) is also sufficiently new to reach a reliable conclusion. To further assess the generalizability of our findings based on Asian individuals, we also included the European population (11,590 cases and 110,189 controls) from the FinnGen database (https://storage.googleapis.com/finngen-public-data-r8/summary_stats). The above data was publicly available information and additional ethical approval was not required. Our study was approved by the Ethical Committee of Shanghai Tenth People’s Hospital.

### MR analysis

Three common MR methods [MR Egger, weighted median, and inverse variance weighted (IVW)] were used to investigate the causal relationships between exposure and outcome.The primary analytical method is IVW method because it provides more accurate and reliable estimation results (13). When heterogeneity or pleiotropy exists, other MR methods were applied to correct for the bias (14). Sensitivity analysis included three primary methods: (1) Cochrans Q-statistic was applied to detect the heterogeneity; (2) MR Egger intercept test was applied to detect the pleiotropy; (3) The leave-one-out method was applied to detect the presence of outlier SNPs. “TwoSampleMR” (version 0.5.7) was the primary R package and all the statistical analyses were done in R software (version 4.3.1). P-value less than 0.05 was considered statistically significant.

## Results

### Causal relationships between gut microbiota and prostate cancer

**Figure 2:**
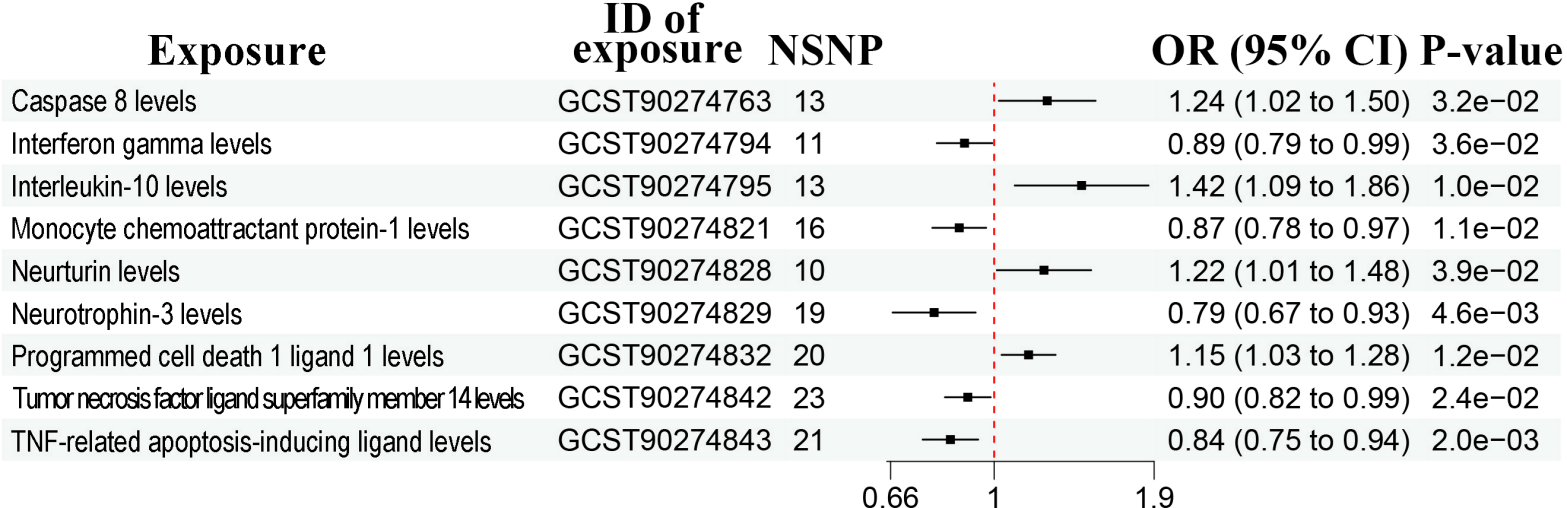
Forest plots showing causal relationships between gut microbiota and prostate cancer. An OR value below 1 (red dashed line) indicates a protective effect, while an OR value above 1 indicates an increased risk. NSNP, number of single nucleotide polymorphisms; OR, odds ratio; 95% CI, 95% confidence interval.

The sample sizes were 1,531-14,306 and 114,755 (5408 cases and 109,347 controls) in the exposure and outcome dataset, respectively (**Table 1**). According to the IVW method, there were a total of 17 bacterial traits to be significantly associated with prostate cancer (**Figure 2**, **Supplementary Figure 1**, P < 0.05). Detailed list of causal relationships between each bacterial trait and prostate cancer was provided in **Supplementary Table 1**. Out of these 17 traits, five were negatively associated with prostate cancer, and the causal relationship between *F_Peptostreptococcaceae* (ebi-a-GCST90016946) and prostate cancer was further confirmed by the weighted median method (**Supplementary Table 1**, OR = 0.70, 95% CI = 0.51-0.98, P = 0.035). Nine bacterial traits were positively associated with prostate cancer, and three were further confirmed by the weighted median method (**Supplementary Table 1**), including *G_Erysipelotrichaceae* UCG003 (ebi-a-GCST90016994, OR = 1.81, 95% CI = 1.06-3.09, P = 0.030), *G_Gordonibacter* (ebi-a-GCST90027692, OR = 1.20, 95% CI = 1.01-1.42, P = 0.037), and *S_Gordonibacter_pamelaeae* (ebi-a-GCST90027761, OR = 1.20, 95% CI = 1.00-1.43, P = 0.044).

**Table 1:** Basic information of exposure and outcome.

Exposure/outcome	Sample size	Ancestry	Year	NSNP	Data linkage
Gut microbiota	1,531-14,306	European	2021-2022	335,714 – 5,729,268	https://mibiogen.gcc.rug.nl
Metabolites	231-7,822	European	2014	2,531,353 – 2,546,774	https://www.nature.com/articles/ng.2982
Cytokines	14,824	European	2023	/	https://www.ncbi.nlm.nih.gov/pmc/articles/PMC10457199/
Prostate cancer	114,755	East Asian	2019	8,878,753	https://gwas.mrcieu.ac.uk/datasets/bbj-a-148/

NSNP, number of single nucleotide polymorphisms.

The heterogeneity test and MR-Egger regression (MR-Egger) showed no evidence of heterogeneity and pleiotropy (**Supplementary Table 2**, P > 0.05). The leave-one-out method suggested no outliers (**Supplementary Figure 2**).

### Causal relationships between metabolites and prostate cancer

**Figure 3:**
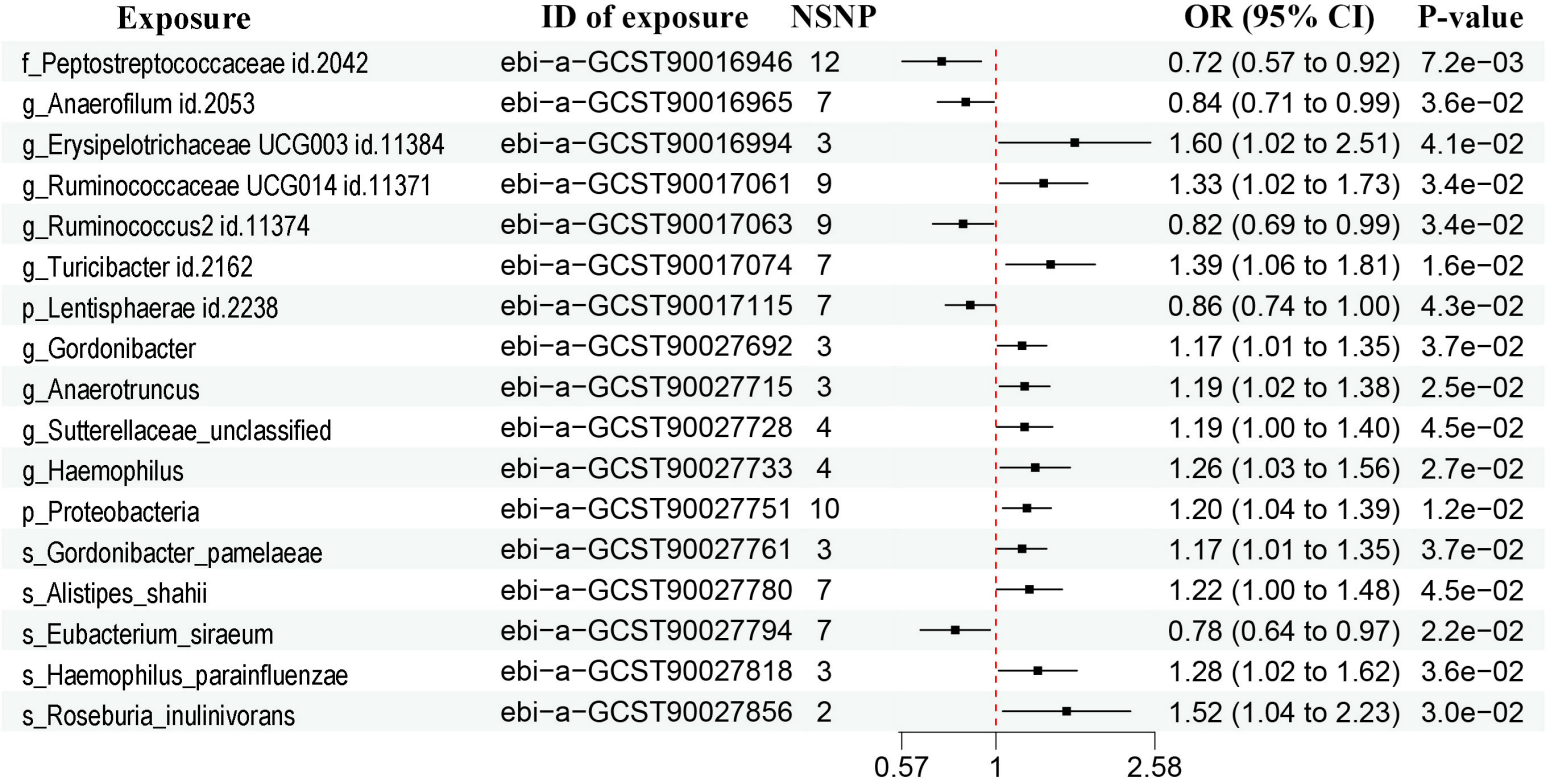
Forest plots showing causal relationships between metabolites and prostate cancer. An OR value below 1 (red dashed line) indicates a protective effect, while an OR value above 1 indicates an increased risk. NSNP, number of single nucleotide polymorphisms; OR, odds ratio; 95% CI, 95% confidence interval.

A total of 452 metabolites were included for MR analysis and there were 28 metabolites significantly associated with prostate cancer according to the IVW method (**Figure 3**, **Supplementary Figure 3**). Detailed list of causal relationships between each metabolite and prostate cancer was provided in **Supplementary Table 3**. We extracted 81 gut microbiota-associated metabolites in the Human Metabolome Database (HMDB) (**Supplementary Table 4**). Out of these 28 metabolites, two gut microbiota-associated metabolites (phenyllactate and serum total cholesterol) were shown to be positively associated with prostate cancer (**Figure 3**, phenyllactate, OR = 2.42, 95% CI = 1.26-4.66, P = 0.008; cholesterol, OR = 1.15, 95% CI = 1.02-1.30). The Q test revealed no heterogeneity (**Supplementary Table 2**, phenyllactate, Q = 9.640, P = 0.788; cholesterol, Q = 18.600, P = 0.885). MR-Egger regression also revealed no pleiotropy (**Supplementary Table 2**, phenyllactate, Egger intercept = 0.010, P = 0.592; cholesterol, Egger intercept = 0.016, P = 0.111). The leave-one-out analysis indicated no outliers (**Supplementary Figure 4**).

### Causal relationships between cytokines and prostate cancer

**Figure 4:**
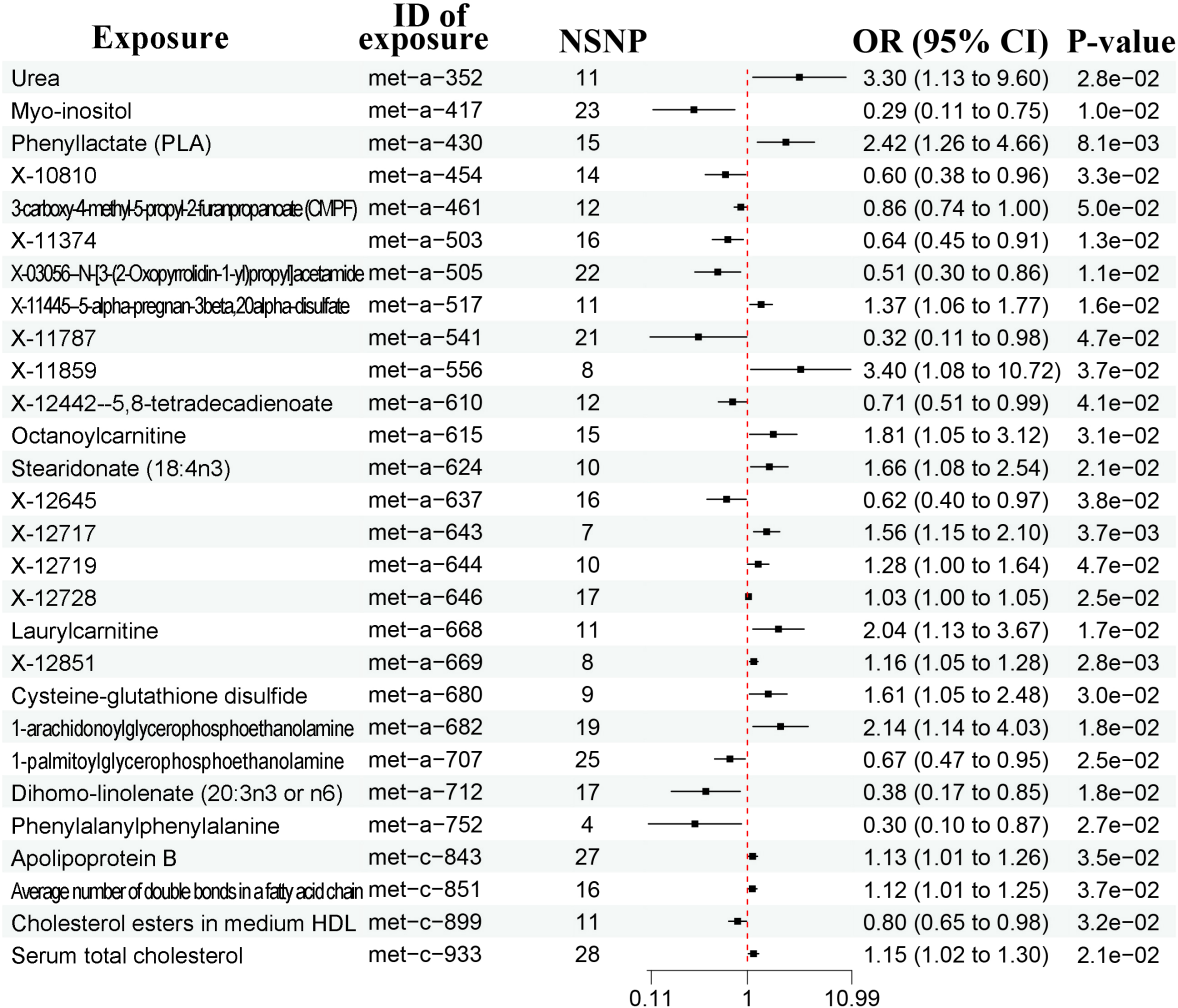
Forest plots showing causal relationships between cytokines and prostate cancer. An OR value below 1 (red dashed line) indicates a protective effect, while an OR value above 1 indicates an increased risk. NSNP, number of single nucleotide polymorphisms; OR, odds ratio; 95% CI, 95% confidence interval.

A total of 91 cytokines were included for MR analysis and there were nine significantly associated with prostate cancer according to the IVW method (**Figure 4**, **Supplementary Figure 5**). Detailed list of causal relationships between each cytokine and prostate cancer was provided in **Supplementary Table 5**. Out of these 9 cytokines, four were positively associated with prostate cancer, while five were negatively associated. In particular, the causal effect of three cytokines were further confirmed by the weighted median method [**Supplementary Table 5**, monocyte chemoattractant protein-1 levels, OR = 0.85, 95% CI = 0.74-0.98, P = 0.027; TNF-related apoptosis-inducing ligand levels (TNFSF10), OR = 0.81, 95% CI = 0.68-0.95, P = 0.009; tumor necrosis factor ligand superfamily member 14 levels (TNFRSF14); OR = 0.87, 95% CI = 0.77-0.99, P = 0.043].

There were no evidence of heterogeneity and pleiotropy (**Supplementary Table 2**, P > 0.05). No outliers existed based on the leave-one-out method (**Supplementary Figure 6**).

### Causal relationships between gut microbiota, metabolites, and cytokines

Considering the potential causal effects of gut microbiota on metabolites and cytokines, we included the aforementioned positive results to perform MR analyses. There were five bacterial traits significantly associated with cytokines (**Table 2**). However, ebi-a-GCST90027780 (*S_Alistipes_shahii*) was negatively associated with GCST90274763 (caspase 8 levels). Given the positive relationship between caspase 8 and prostate cancer, *S_Alistipes_shahii s*hould be associated with a decreased risk of prostate cancer, which contradicted the MR results between gut microbiota and prostate cancer (**Figure 2**). Therefore, *S_Alistipes_shahii*/caspase 8/prostate cancer may be not a reliable causal inference. The other four causal inferences were consistent with the MR results between gut microbiota and prostate cancer: *G_Ruminococcus2* (ebi-a-GCST90017063)/neurturin levels (NTRN, GCST90274828)/prostate cancer, *G_Anaerofilum* (ebi-a-GCST90016965)/TNFRSF14 (GCST90274842)/prostate cancer, *G_Ruminococcaceae UCG014* (ebi-a-GCST90017061)/TNFSF10 (GCST90274843)/prostate cancer, and *G_Erysipelotrichaceae UCG003* (ebi-a-GCST90016994)/TNFSF10 (GCST90274843)/prostate cancer. Additionally, ebi-a-GCST90027751 (P_Proteobacteria) was positively with met-c-933 (serum total cholesterol), which was consistent with both the positive causal effect of cholesterol on prostate cancer and the positive causal effect of P_Proteobacteria on prostate cancer (**Table 2**).

**Table 2:** Causal effects of gut microbiota on metabolites and cytokines.

Exposure	ID.exposure	Outcome	ID.outcome	NSNP	Beta	SE	OR	95% CI	P-value
*s_Alistipes_shahii*	ebi-a-GCST90027780	Caspase 8 levels	GCST90274763	11	-0.100	0.047	0.905	0.825-0.992	0.034
*g_Ruminococcus2 id.11374*	ebi-a-GCST90017063	Neurturin levels	GCST90274828	15	-0.123	0.060	0.885	0.786-0.995	0.041
*g_Anaerofilum id.2053*	ebi-a-GCST90016965	Tumor necrosis factor ligand superfamily member 14 levels	GCST90274842	11	0.093	0.043	1.097	1.009-1.193	0.030
*g_Ruminococcaceae UCG014 id.11371*	ebi-a-GCST90017061	TNF-related apoptosis-inducing ligand levels	GCST90274843	11	-0.132	0.061	0.877	0.778-0.988	0.031
*g_Erysipelotrichaceae UCG003 id.11384*	ebi-a-GCST90016994	TNF-related apoptosis-inducing ligand levels	GCST90274843	4	-0.215	0.107	0.807	0.653-0.996	0.046
p_Proteobacteria	ebi-a-GCST90027751	Serum total cholesterol	met-c-933	11	0.117	0.037	1.124	1.045-1.209	0.002

NSNP, number of single nucleotide polymorphisms; OR, odds ratio; 95% CI, 95% confidence interval; SE, standard error.

### Assessment of the generalizability of our conclusions

The aforementioned positive findings regarding gut microbiota, metabolites, and cytokines were treated as exposures, while European individuals were considered outcomes. We further performed MR analyses to assess the generalizability of our conclusions. As shown in **Supplementary Table 6**, a few gut microbiota and metabolites were significantly associated with prostate cancer in both Asian and European population.

## Discussion

Using large GWAS summary datasets, we found that 17 bacterial traits, 28 metabolites, and 9 cytokines had causal relationships with prostate cancer in Asians. As is well known, gut microbiota also affects the occurrence and development of malignancies via metabolites and cytokines (15, 16). Further MR analyses indicated that *G_Ruminococcus2*/NTRN/prostate cancer, *G_Anaerofilum*/TNFRSF14/prostate cancer, *G_Ruminococcaceae UCG014*/TNFSF10/prostate cancer, and *G_Erysipelotrichaceae UCG003*/TNFSF10/prostate cancer, and P_Proteobacteria/cholesterol/prostate cancer were potential protective or risk signaling pathways. These findings suggest potential therapeutic targets for prostate cancer in Asia. However, given the significant genetic disparities in prostate cancer between Asian and European populations, the generalizability of our conclusions may be limited within Europeans. Studies have shown that Asian populations exhibit higher mutation rates of SPOP and FOXA1 in prostate cancer, while European populations are more characterized by ERG fusion mutations and PTEN loss (17). Even for the same SNP (e.g. ESR2 rs1256049), its association with prostate cancer susceptibility may exhibit opposite effects across different ethnic populations (18). These inter-ethnic genetic differences may also explain why only a small subset of gut microbiota and metabolites showed significant causal relationships with prostate cancer in both Asian and European populations according to our findings.

Both F_Peptostreptococcaceae and *G_Anaerofilum* were protective factors based on our analysis. These results were consistent with the study conducted by Makoto et al. (9), who identified a cluster of gut microbiota including the two bacteria negatively associated with high-risk prostate cancer. However, they showed that low-risk prostate cancer shared similar bacterial abundance with healthy control population, which may be caused by indolent biological behavior of low-risk prostate cancer (19). In another fecal microbiome analysis of 64 prostate cancer patients (20), Liss et al. reported that the abundance of Bacteroides and Streptococcus species was significantly different in prostate cancer from those without cancer, which was not in accord with our findings. A possible explanation for the inconsistency may be the small sample size. Increasing the sample size may make the findings tend to be consistent. Additionally, our analysis indicated that *G_Anaerofilum* may decrease the risk of prostate cancer via TNFRSF14 pathway. TNFRSF14, also called CD270 or herpes virus entry mediator (HVEM), is an immune checkpoint regulatory molecule and mainly expressed on various immune cells (21). It played a crucial role in the tumorigenesis, tumor development, and immunotherapy of a variety of malignancies, including prostate cancer (22, 23). Prostate cancer is usually considered as a “cold tumor”, which does not respond favorably to immunotherapy (24). *G_Anaerofilum*/TNFRSF14 axis might serve as a promising target for immunotherapy in prostate cancer.

Proteobacteria is a facultative anaerobe and generally predicts the presence of dysbiosis (25). In prostate cancer, Proteobacteria was significantly positively associated with lymph node metastasis and distant metastasis (26), which was consistent with our results of the positive causal relationship between P_Proteobacteria and prostate cancer. Our data also demonstrated that P_Proteobacteria may promote the progression of prostate cancer by regulating cholesterol metabolism. High cholesterol levels were tightly related to an increased risk of prostate cancer, which has been demonstrated by considerable epidemiological evidence (27). A randomized controlled trial showed that lifestyle factors (e.g. high-fat diet and obesity), well established risk factors for prostate cancer, might influence the progression of prostate cancer through gut microbiota including Proteobacteria (28). These findings largely supported our conclusions of the P_Proteobacteria/cholesterol/prostate cancer axis. However, more clinical and laboratory data are needed for confirmation.

There were a few limitations in the study. First, there were ethnic discrepancies between the exposure and outcome, which might bring a selection bias. However, the bias seemed clinically acceptable as our MR results were partially consistent with previous studies, including extensive 16S rRNA and metagenomic sequencing data. Second, this was short of experimental validation although we identified several signaling pathways involved in prostate cancer. Third, we performed the study using the latest and most comprehensive GWAS summary datasets of bacterial traits, metabolites, and cytokines. Nevertheless, the data depth and breadth were still relatively limited to reach more reliable and stable conclusions.

## Conclusion

In summary, we found that there were causal associations of gut microbiota, metabolites, and cytokines with prostate cancer in Asians. The causal effects of gut microbiota on prostate cancer were partially mediated by metabolites and cytokines. These findings might contribute to the development of new therapeutic strategies for prostate cancer.

## Data Availability

The original contributions presented in the study are included in the article/**Supplementary Material**. Further inquiries can be directed to the corresponding authors.

## Supplementary material

Supplementary Figure 1 Scatter plots showing causal relationships between gut microbiota and prostate cancer.

Supplementary Figure 2 Leave-one-out sensitivity analysis for causal relationships between gut microbiota and prostate cancer.

Supplementary Figure 3 Scatter plots showing causal relationships between metabolites and prostate cancer.

Supplementary Figure 4 Leave-one-out sensitivity analysis for causal relationships between gut microbiota-associated metabolites and prostate cancer.

Supplementary Figure 5 Scatter plots showing causal relationships between cytokines and prostate cancer.

Supplementary Figure 6 Leave-one-out sensitivity analysis for causal relationships between cytokines and prostate cancer.

Supplementary Table 6 Gut microbiota, metabolites, and cytokines that had significant causal relationships with prostate cancer in both Asian and European populations.
