# Correction: Gut microbiota, metabolites, and cytokines in relation to the risk of prostate cancer in the Asian population

**DOI:** 10.3389/fonc.2025.1647090

**Published:** 2025-07-01

**Authors:** Zhengshi Wang, Haotian Chen, Yongqiang Liu, Libin Zou, Zhijin Zhang, Zhiqiang Yin, Shiyu Mao, Changcheng Guo, Bin Yang, Pengfei Wu, Xudong Yao

**Affiliations:** ^1^ Department of Urology, Shanghai Tenth People’s Hospital, Clinical Medical College of Nanjing Medical University, Shanghai, China; ^2^ Department of Urology, Shanghai Tenth People’s Hospital, School of Medicine, Tongji University, Shanghai, China; ^3^ Urologic Cancer Institute, School of Medicine, Tongji University, Shanghai, China; ^4^ Department of Breast and Thyroid Surgery, Shanghai Tenth People’s Hospital, Tongji University School of Medicine, Shanghai, China; ^5^ Shanghai Center of Thyroid Diseases, Shanghai Tenth People’s Hospital, Tongji University School of Medicine, Shanghai, China

**Keywords:** prostate cancer, GWAS, gut microbiota, single nucleotide polymorphism, cytokine

In the published article, there was an error regarding the affiliations for Xudong Yao. As well as having affiliation(s) 2, 3, they should also have “Department of Urology, Shanghai Tenth People’s Hospital, Clinical Medical College of Nanjing Medical University, Shanghai, China”.

The authors apologize for this error and state that this does not change the scientific conclusions of the article in any way. The original article has been updated.

