# Supplementary figures and images for: Gut microbiota, metabolites, and cytokines in relation to the risk of prostate cancer in the Asian population
Source: Front Oncol. 2025 Jan 15;14:1466190. doi: 10.3389/fonc.2024.1466190 (PMC11774728; doi:10.3389/fonc.2024.1466190)

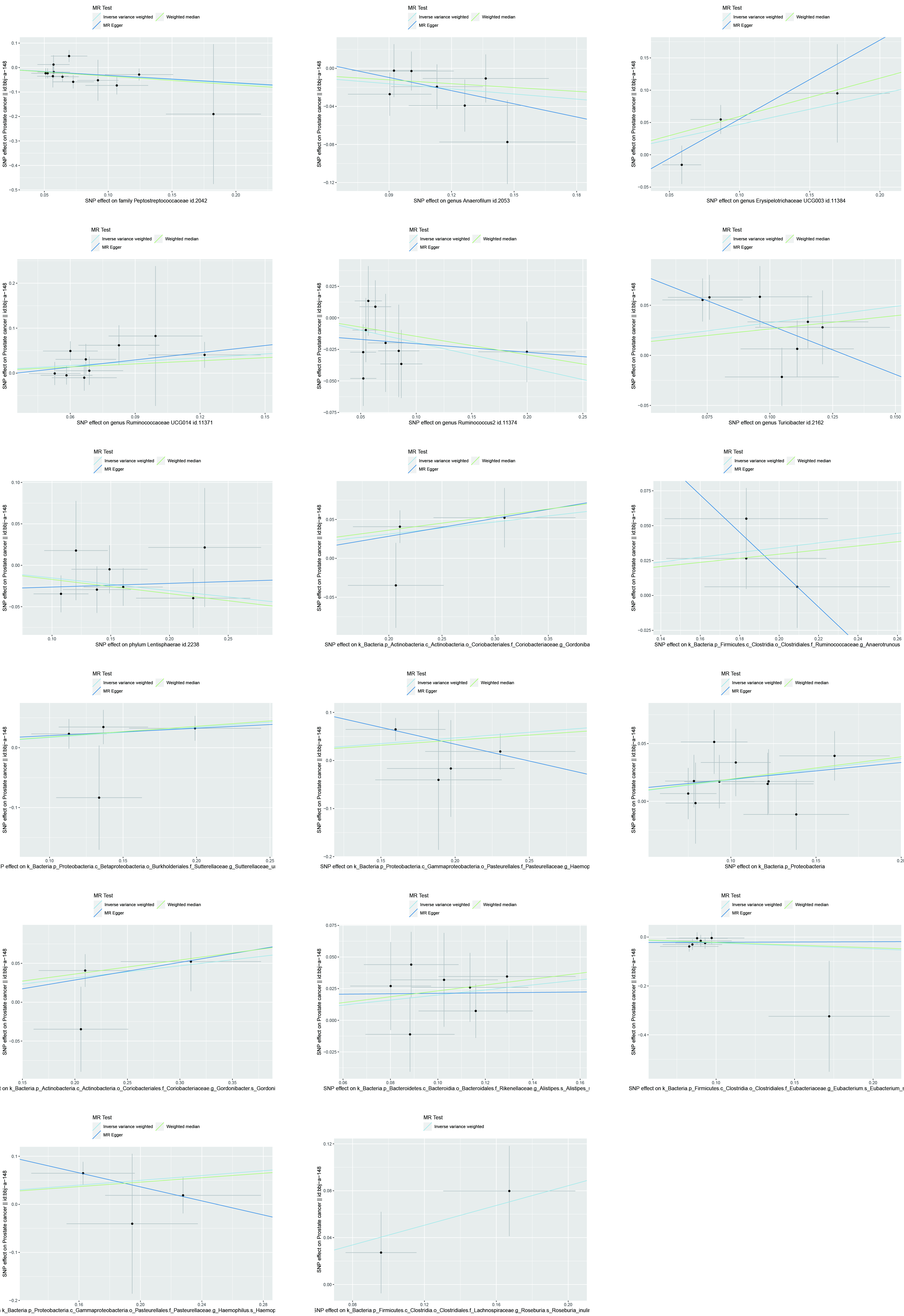

Supplement: Supplementary Figure 1 — Scatter plots showing causal relationships between gut microbiota and prostate cancer. [file Image1.jpeg]

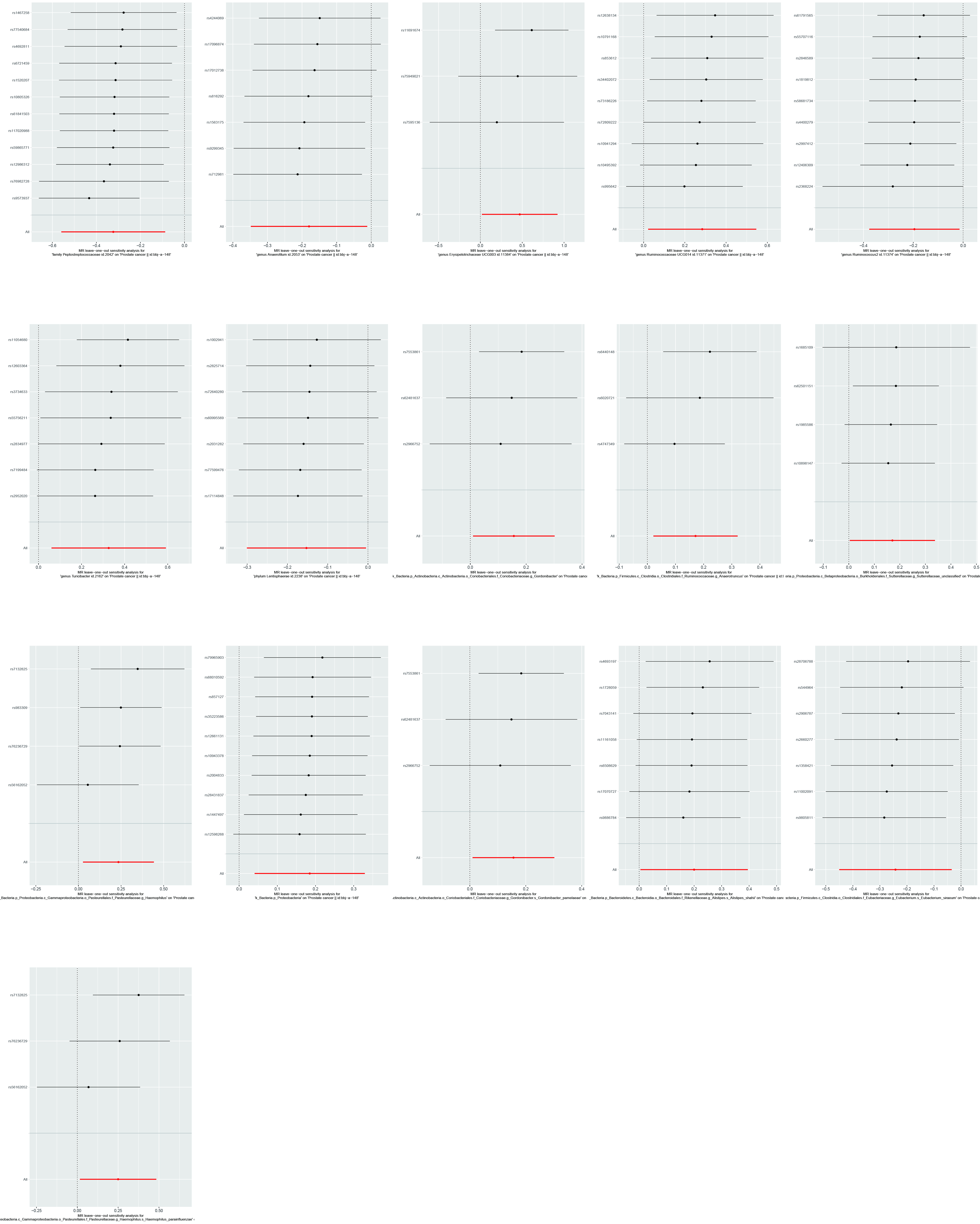

Supplement: Supplementary Figure 2 — Leave-one-out sensitivity analysis for causal relationships between gut microbiota and prostate cancer. [file Image2.jpeg]

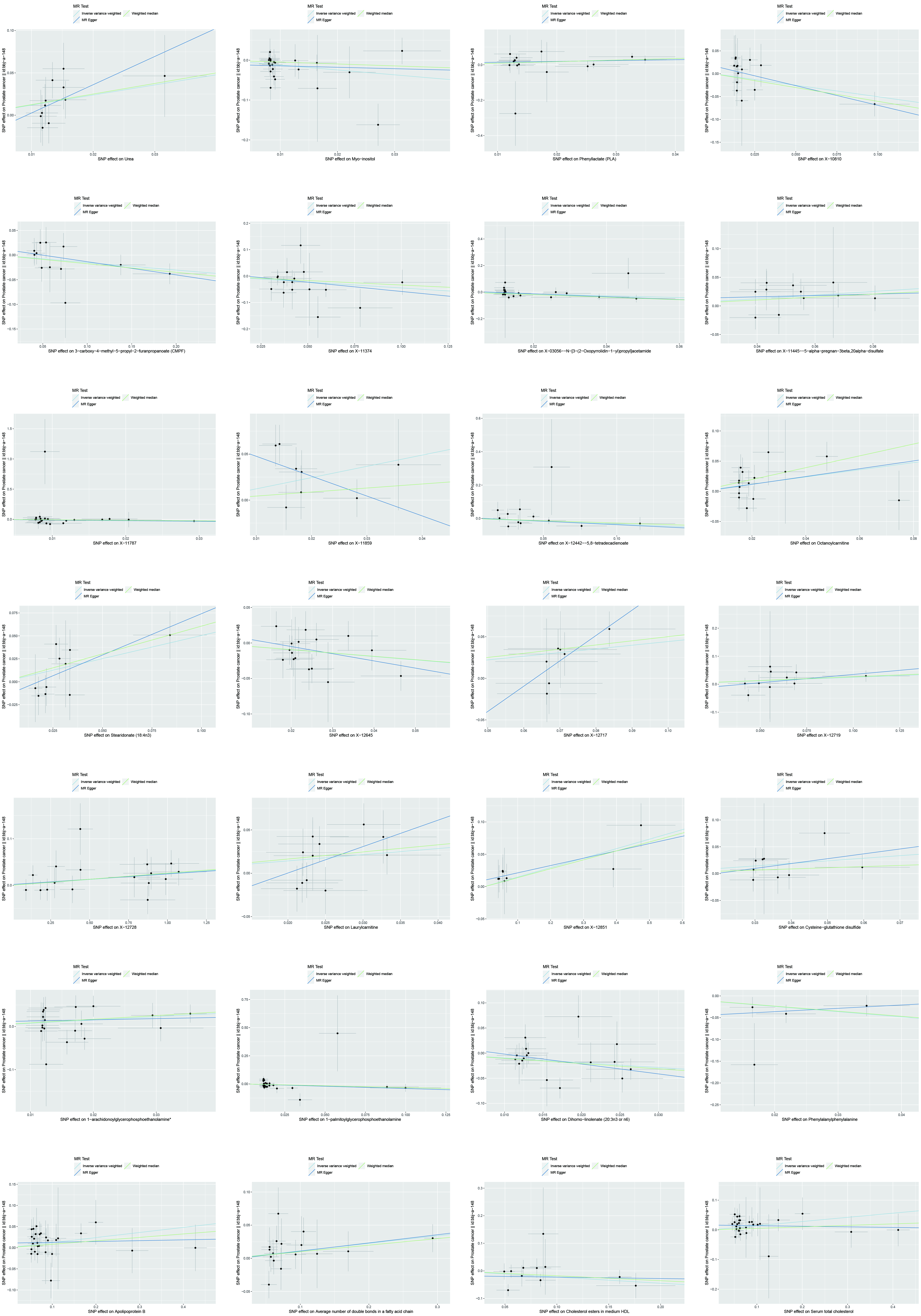

Supplement: Supplementary Figure 3 — Scatter plots showing causal relationships between metabolites and prostate cancer. [file Image3.jpeg]

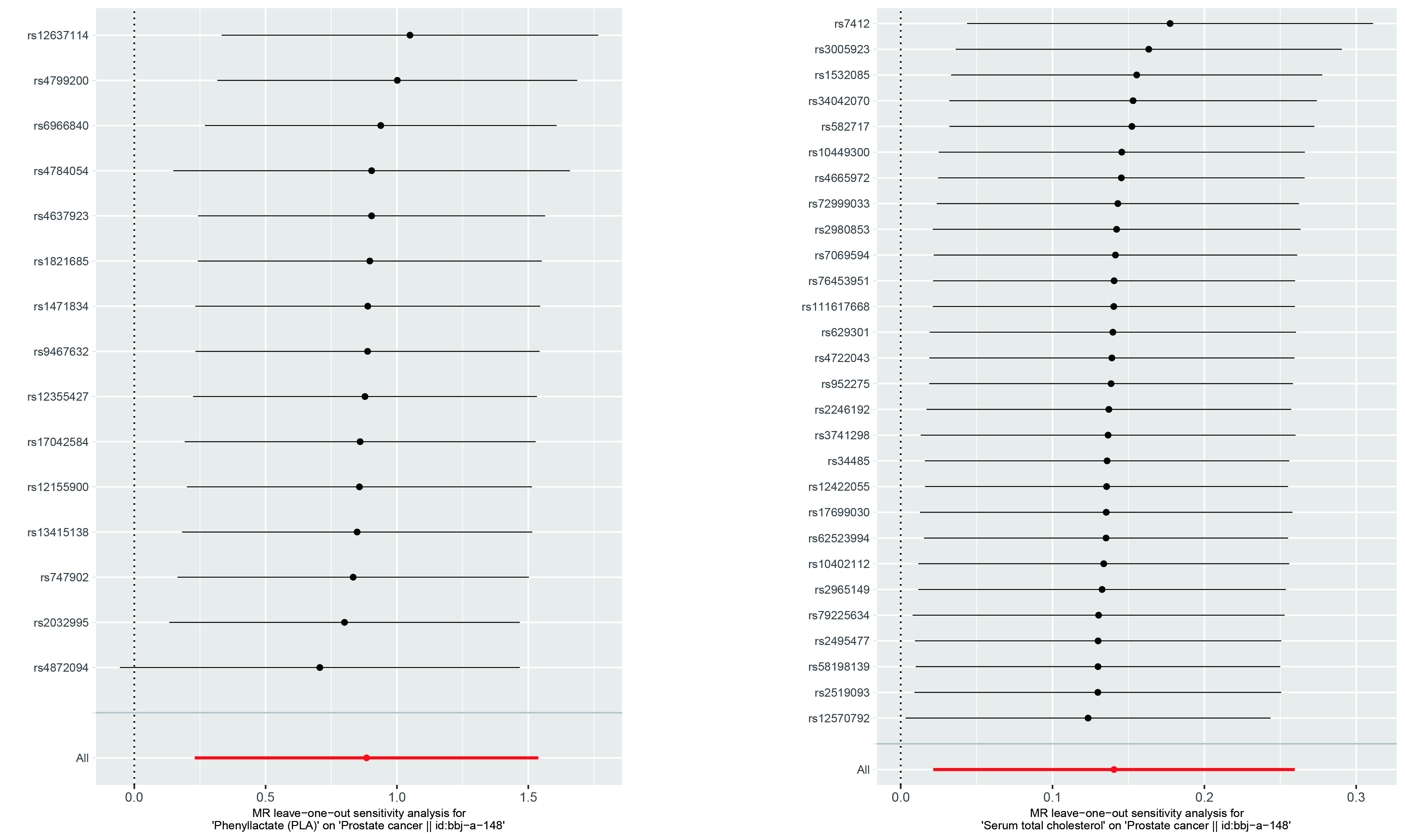

Supplement: Supplementary Figure 4 — Leave-one-out sensitivity analysis for causal relationships between gut microbiota-associated metabolites and prostate cancer. [file Image4.jpeg]

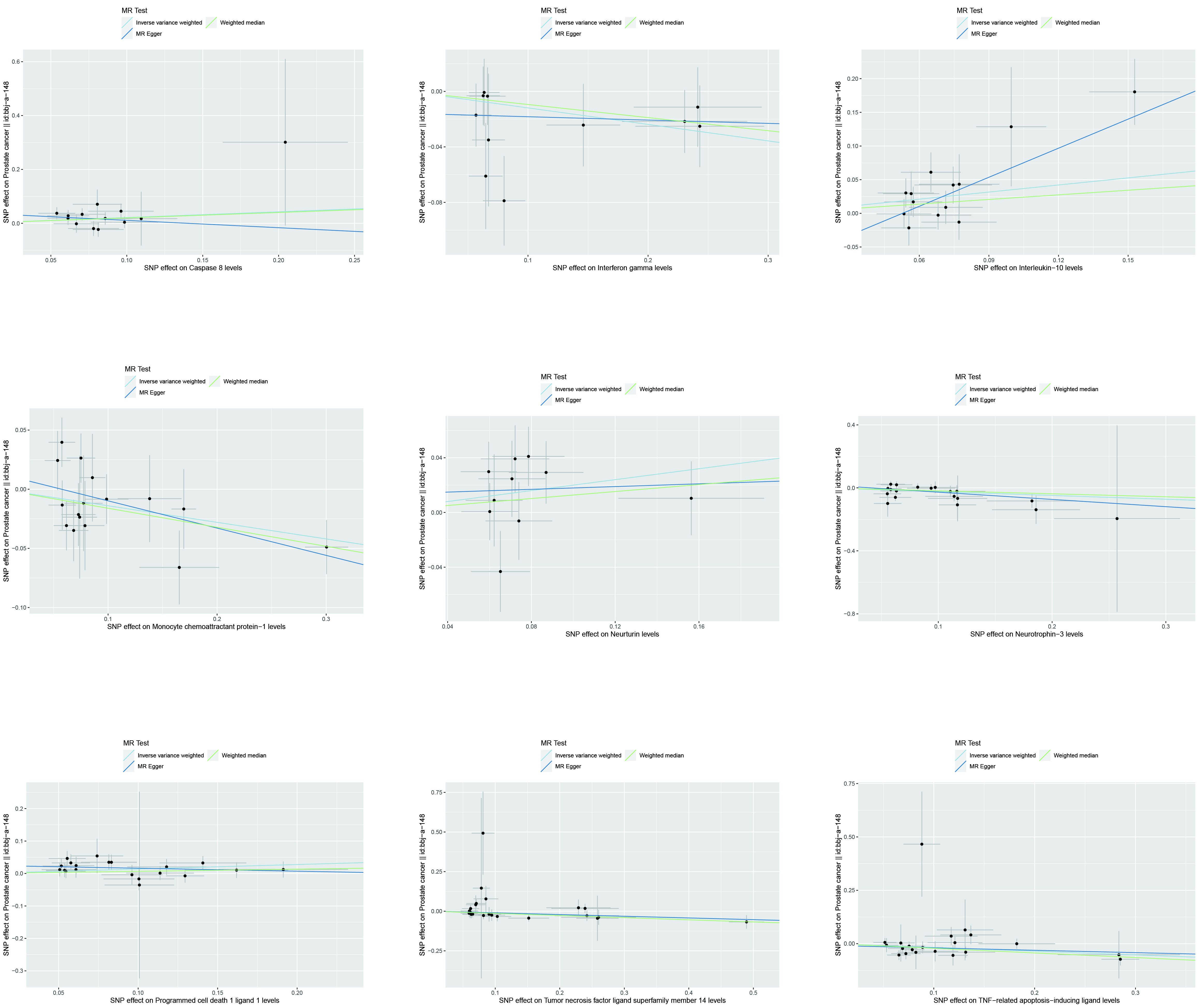

Supplement: Supplementary Figure 5 — Scatter plots showing causal relationships between cytokines and prostate cancer. [file Image5.jpeg]

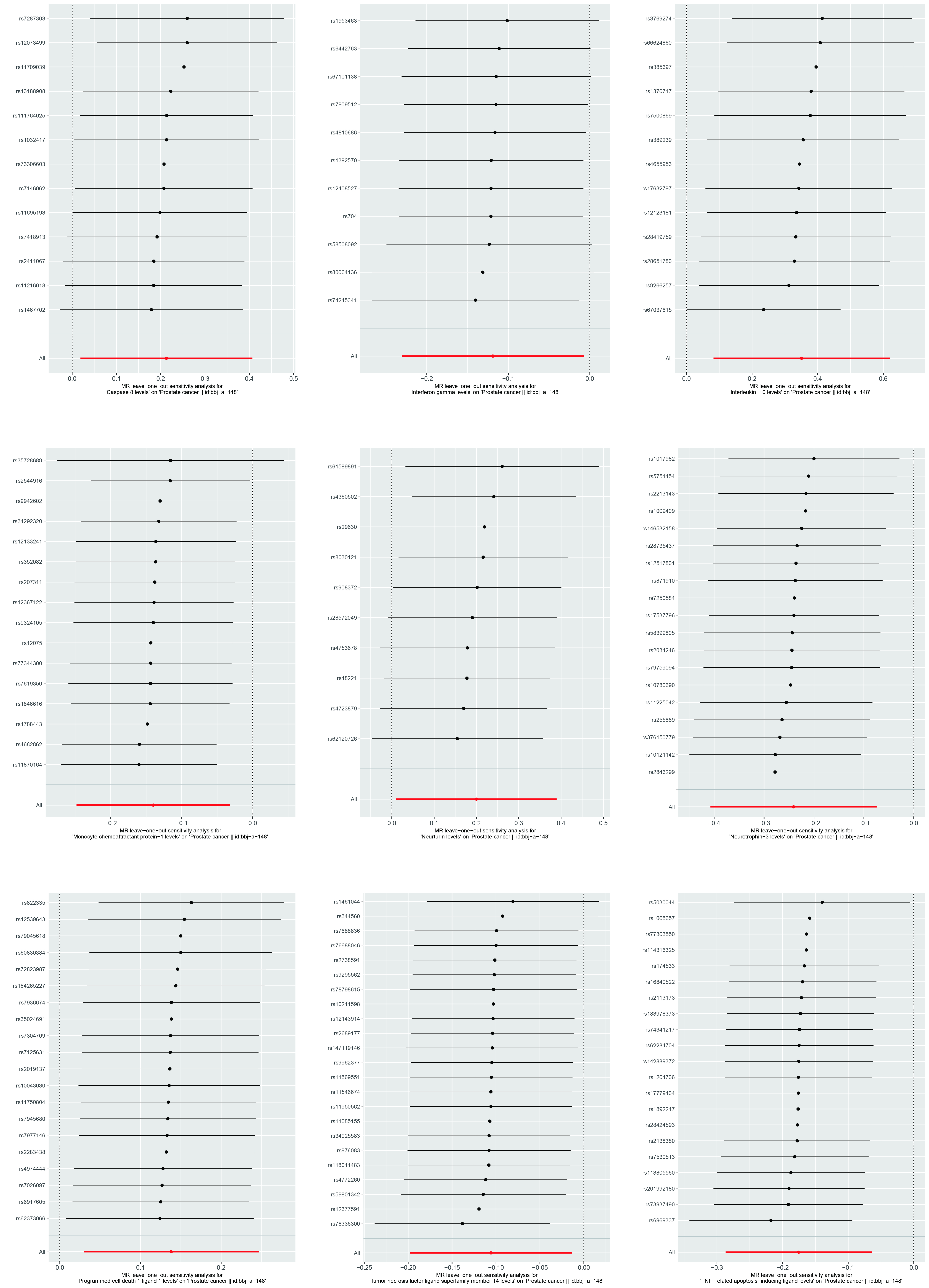

Supplement: Supplementary Figure 6 — Leave-one-out sensitivity analysis for causal relationships between cytokines and prostate cancer. [file Image6.jpeg]
